# AMP-protein kinase may protect against sepsis-induced acute kidney injury through modulation of immune response and endothelial activation

**DOI:** 10.1186/cc13425

**Published:** 2014-03-17

**Authors:** H Gomez, D Escobar, A Botero, BS Zuckerbraun

**Affiliations:** 1 University of Pittsburgh, PA, USA

## Introduction

Organ injury is a hallmark of sepsis, in particular acute kidney injury (AKI). Yet the mechanisms involved in sepsis-induced AKI are not well understood. Energy prioritization is an important cell defense mechanism, and thus we hypothesized that exogenous activation of AMP-protein kinase (AMPK), a master regulator of cellular energy metabolism, protects against sepsis-induced AKI.

## Methods

Sixty C57BL/6 male mice, 6 to 8 weeks of age, weighing 20 to 25 g were divided into six groups: 1, cecal ligation and puncture (CLP); 2, CLP+AICAR (AI, AMPK activator, 100 mg/kg 24 hours before CLP); 3, CLP+compound C (AMPK inhibitor, 30 mg/kg; CoC); 4, sham; 5, sham+AI; 6, sham+CoC. Blood/tissue samples were collected 8 hours after CLP. Renal function (creatinine (Cr, mg/dl), BUN (mg/dl) and cystatin C (CysC, ng/ml)), cytokine expression (ELISA), endothelial activation (IcAm- 1 expression), neutrophil adhesion (PMN, fluorescence, anti-CD11b mAb-tagged PMNs) and vascular leak (Evan's blue) were assessed. The effect of AI given 4 hours (*n *= 12) before and 2 hours after (*n *= 11) CLP was also evaluated. The experiment was reproduced in vitro using cell culture (renal epithelial, endothelial cells and macrophages), with similar groups: 1, control; 2, control+AI (1 mM/1 hour pre LPS); 3, LPS (100 ng/ml for 4 hours); 4, LPS+AI; 5, control+CoC (10 μM/1 hour pre LPS); 6, LPS+CoC. Cytokines were measured with ELISA. Data are presented as mean ± SD and as LPS/CLP and LPS/CLP+AI.

## Results

AI prevented Cr, BUN and CysC from increasing after CLP (0.43 ± 0.18, 0.2 ± 0.02, *P *= 0.0003; 62.2 ± 10.8, 43.7 ± 17.7, *P *= 0.01; 103.9 ± 54.8, 49.3 ± 30.8, *P *= 0.01, respectively). AICAR decreased cytokine release after CLP (IL-6: 1,818 ± 344 vs. 1,374 ± 268, *P *= 0.04; IL- 10: 7,592 ± 5,038 vs. 2,245 ± 2,668, *P *= 0.05; TNFα: 213 ± 172 vs. 39 ± 16, *P *= 0.03) and LPS in macrophage culture (IL-6: 241.7 ± 1.5 vs. 7.2 ± 1.5, *P *= 0.055; TNF: 1,882 ± 583 vs. 82 ± 28, *P *= 0.04). However, AI lost its protective signal when administered 4 hours pre or 2 hours after CLP (Cr CLP vs. CLP+AI: 0.2 vs. 0.2, *P *= 0.1 and 0.45 ± 0.3 vs. 0.56 ± 0.3, *P *= 0.5, respectively). AI also decreased ICAM-1 expression in vivo and in vitro, vascular leak (0.09 ± 0.01 vs. 0.06 ± 0.008, *P *= 0.003) and PMN adhesion (1,055 ± 179 vs. 751 ± 242, *P *= 0.04).

## Conclusion

AMPK activation by AICAR prevented sepsis-induced AKI. AICAR decreased cytokine release, endothelial activation and vascular leak, suggesting that organ protection may be mediated by modulation of the inflammatory response and protection of the microvasculature. However, AICAR's protective effect may be conditional to timing of administration.

